# Editorial: Host-Guest Chemistry of Macrocycles

**DOI:** 10.3389/fchem.2020.628200

**Published:** 2020-12-09

**Authors:** Tangxin Xiao, Robert Elmes, Yong Yao

**Affiliations:** ^1^School of Petrochemical Engineering, Changzhou University, Changzhou, China; ^2^Department of Chemistry, Maynooth University, National University of Ireland, Maynooth, Ireland; ^3^Synthesis and Solid-State Pharmaceutical Centre, Maynooth University, National University of Ireland, Maynooth, Ireland; ^4^School of Chemistry and Chemical Engineering, Nantong University, Nantong, China

**Keywords:** macrocycles, supramolecular chemistry, host-guest interaction, self-assembly, pillar[n]arenes

Macrocycle-based host–guest chemistry has played an important role in the development of supramolecular chemistry. Burgeoning progress has been continuously made in the development of supramolecular assemblies by using various host molecules, such as cyclodextrins (Li and Purdy, 1992; Szejtli, 1998; Harada et al., 2009, 2014; Antoniuk and Amiel, 2016), cucurbiturils (Lagona et al., 2005; Ni et al., 2014; Barrow et al., 2015; Murray et al., 2017), calixarenes (Shinkai et al., 1984; Böhmer, 1995; Guo and Liu, 2014), crown ethers (Pedersen, 1967; Amabilino et al., 1995; Zhang et al., 2007; Xiao et al., 2020a, 2021), pillararenes (Ogoshi et al., 2008, 2016, 2018; Xue et al., 2012; Strutt et al., 2014; Kakuta and Yamagishi, 2018; Xiao et al., 2018, 2019a,b,c), and other macrocycles (Wang et al., 2019). The architectures and properties of different hosts endow themselves with versatile abilities to bind with different guest molecules. Therefore, macrocyclic hosts show significant potential in constructing assorted functional materials, such as hydrogels (Appel et al., 2012; Xiao et al., 2019d), functional supramolecular polymers (Chen et al., 2019; Xiao et al., 2020b), artificial light harvesting systems (Xiao et al., 2019e), and so on.

Supramolecular polymers, a concept combining elements of both supramolecular chemistry and polymer science, are promising dynamic functional materials. Moreover, the incorporation of fluorophores into supramolecular polymers could endow them with interesting photophysical properties. In this context, Zhang et al. reviewed fluorescent supramolecular polymers constructed by crown ether-based host-guest interactions. They focused on fabrication strategies, properties, and potential applications of these materials. On the same Topic, Wu and Xiao contributed an article on an aggregation induced emissive (AIE) supramolecular polymer which was constructed from a cyanostilbene based ditopic benzo-21-crown-7 and a ditopic dialkylammonium salt. In another review paper, Gatiatulin et al. summarized the possible alternatives to the classical key-to-lock principle with higher selectivity for molecular recognition. These alternatives are based on cooperativity of phase transitions, which adds up the small differences in molecular structure of different bound guests. In a minireview, Duan et al. (b) summarized broad approaches for the preparation of graphene nanomaterials functionalized with calix[n]arene/pillar[n]arene, and their applications in molecular recognition, fluorescent sensors, electrochemical biosensors, and as catalytic, antibacterial, and adsorption materials.

Calixarene derivatives have played an important role in developing anti-tumor agents. The contribution of An et al. focused on novel dihomooxacalix[4]arene-based anti-tumor agents. In their work, they reported the synthesis of 19 structurally related dihomooxacalix[4]arene amide derivatives in search of optimal efficacy. Guo et al. synthesized a *p-tert*-butyldihomooxacalix[4]arene, which could form a soft gel in cyclohexane. Moreover, the xerogel with its highly interconnected and homogeneous porous network, may be used for drug storage and controlled release. Pillararenes are a relatively new macrocyclic host and have been employed to fabricate various supramolecular materials. Macrocyclic amphiphiles have attracted much attention due to their unique properties in the construction of functional nanomaterials. The work of Wang et al. investigates a pillararene-based macrocyclic amphiphile, which is responsive to pH. Interestingly, a pH-induced transition between single-chain macrocyclic amphiphile and bola-type amphiphile and the corresponding self-assembly behavior was investigated. Self-assembled peptides can also be used to fabricate new biomaterials for medical applications. Duan et al. (a) developed cationic pillar[6]arene-modified graphene films on glassy carbon electrodes directly from graphene oxide-cationic pillar[6]arene dispersions using a pulsed electrodeposition technique. Experimental results revealed that the electrochemically reduced graphene oxide-cationic pillar[6]arene films could show a much higher electrochemical response to five purine bases than unmodified reduced graphene oxide films and bare glassy carbon electrodes. In other work, Duan, Wang, Zhang et al. designed new host-guest binding motifs based on a water-soluble pillar[6]arene dodecyl-ammonium chloride with two aromatic sulfonic acids in water. Interestingly, both of these host-guest complexes can be tuned reversibly between their complexed and decomplexed states by sequential addition of a base and an acid (NaOH and HCl, respectively). Construction of polypseudorotaxanes in high-polar organic solvents is challenging owing to the weak interactions between macrocycles and axles. Su et al. prepared a novel metal-coordinated poly[2]pseudorotaxane by using pillar[5]arene, 1,4-bis(4-pyridyl pyridinium)butane, and [PdCl_2_(PhCN)_2_] in dimethyl sulfoxide.

Chaudhuri et al. developed a method to promote clean and environmentally friendly disinfection of phenolic substrates by employing α-cyclodextrin to affect the product distribution in chlorine dioxide-mediated decomposition of organic pollutants. Chen et al. synthesized and characterized two novel cyclic γ-peptides with hydrophobic inner surfaces, which could self-assemble into stacking nanotubes through intermolecular H-bonds and π-π interactions. Notably, the nanotubes could serve as selective water channels to transport water across the lipid membrane. Metallacycles have obtained great interest in recent years. The contribution of Wu et al. constructed two porphyrin-based organoplatinum(II) metallacycles through coordination-driven self-assembly. Interestingly, these metallacycles could be utilized as catalysts for photo-oxidization with high efficiency. Zhu et al. demonstrated a simple protocol to prepare stimuli-responsive surface-active microcrystalline cellulose particles which are able to reversibly self-assemble at a fluid interface via reversible *in situ* hydrophobization to stabilize stimuli-responsive Pickering emulsions.

In summary, this Research Topic has highlighted how the fields of macrocyclic and supramolecular chemistry are still inextricably linked. The field has matured considerably in recent years and is now beginning to show more and more potential for real-world applications to address important issues across the sciences.

## Author Contributions

All authors listed have made a substantial, direct and intellectual contribution to the work, and approved it for publication.

## Conflict of Interest

The authors declare that the research was conducted in the absence of any commercial or financial relationships that could be construed as a potential conflict of interest. The handling editor declared a past co-authorship with one of the authors RE.

## References

[B1] Amabilino D. B. Ashton P. R. Brown C. L. Cordova E. Godinez L. A. Goodnow T. T. (1995). Molecular meccano. 2. Self-assembly of [n]catenanes. J. Am. Chem. Soc. 117, 1271–1293. 10.1021/ja00109a011

[B2] Antoniuk I. Amiel C. (2016). Cyclodextrin-mediated hierarchical self-assembly and its potential in drug delivery applications. J. Pharm. Sci. 105, 2570–2588. 10.1016/j.xphs.2016.05.010 27342436

[B3] Appel E. A. del Barrio J. Loh X. J. Scherman O. A. (2012). Supramolecular polymeric hydrogels. Chem. Soc. Rev. 41, 6195–6214. 10.1039/C2CS35264H 22890548

[B4] Barrow S. J. Kasera S. Rowland M. J. del Barrio J. Scherman O. A. (2015). Cucurbituril-based molecular recognition. Chem. Rev. 115, 12320–12406. 10.1021/acs.chemrev.5b00341 26566008

[B5] Böhmer V. (1995). Calixarenes, macrocycles with (almost) unlimited possibilities. Angew. Chem. Int. Ed. 34, 713–745. 10.1002/anie.199507131

[B6] Chen Y. Sun S. Lu D. Shi Y. Yao Y. (2019). Water-soluble supramolecular polymers constructed by macrocycle-based host-guest interactions. Chin. Chem. Lett. 30, 37–43. 10.1016/j.cclet.2018.10.022

[B7] Guo D.-S. Liu Y. (2014). Supramolecular chemistry of p-Sulfonatocalix[n]arenes and its biological applications. Acc. Chem. Res. 47, 1925–1934. 10.1021/ar500009g 24666259

[B8] Harada A. Takashima Y. Nakahata M. (2014). Supramolecular polymeric materials via cyclodextrin-guest interactions. Acc. Chem. Res. 47, 2128–2140. 10.1021/ar500109h 24911321

[B9] Harada A. Takashima Y. Yamaguchi H. (2009). Cyclodextrin-based supramolecular polymers. Chem. Soc. Rev. 38, 875–882. 10.1039/B705458K 19421567

[B10] Kakuta T. Yamagishi T.-a. Ogoshi T. (2018). Stimuli-responsive supramolecular assemblies constructed from pillar[n]arenes. Acc. Chem. Res. 51, 1656–1666. 10.1021/acs.accounts.8b00157 29889488

[B11] Lagona J. Mukhopadhyay P. Chakrabarti S. Isaacs L. (2005). The Cucurbit[n]uril family. Angew. Chem. Int. Ed. 44, 4844–4870. 10.1002/anie.200460675 16052668

[B12] Li S. Purdy W. C. (1992). Cyclodextrins and their applications in analytical chemistry. Chem. Rev. 92, 1457–1470. 10.1021/cr00014a009

[B13] Murray J. Kim K. Ogoshi T. Yao W. Gibb B. C. (2017). The aqueous supramolecular chemistry of cucurbit[n]urils, pillar[n]arenes and deep-cavity cavitands. Chem. Soc. Rev. 46, 2479–2496. 10.1039/c7cs00095b 28338130 PMC5462124

[B14] Ni X.-L. Xiao X. Cong H. Zhu Q.-J. Xue S.-F. Tao Z. (2014). Self-assemblies based on the outer-surface interactions of Cucurbit[n]urils: new opportunities for supramolecular architectures and materials. Acc.Chem. Res. 47, 1386–1395. 10.1021/ar5000133 24673124

[B15] Ogoshi T. Kakuta T. Yamagishi T. A. (2018). Applications of pillar[n]arene-based supramolecular assemblies. Angew. Chem. Int. Ed. 58, 2197–2206. 10.1002/anie.201805884 29900642

[B16] Ogoshi T. Kanai S. Fujinami S. Yamagishi T.-A. Nakamoto Y. (2008). Para-bridged symmetrical pillar[5]arenes: their lewis acid catalyzed synthesis and host–guest property. J. Am. Chem. Soc. 130, 5022–5023. 10.1021/ja711260m 18357989

[B17] Ogoshi T. Yamagishi T.-A. Nakamoto Y. (2016). Pillar-shaped macrocyclic hosts pillar[n]arenes: new key players for supramolecular chemistry. Chem. Rev. 116, 7937–8002. 10.1021/acs.chemrev.5b00765 27337002

[B18] Pedersen C. J. (1967). Cyclic polyethers and their complexes with metal salts. J. Am. Chem. Soc. 89, 7017–7036. 10.1021/ja01002a035

[B19] Shinkai S. Mori S. Tsubaki T. Sone T. Manabe O. (1984). New water-soluble host molecules derived from calix[6]arene. Tetrahedron Lett. 25, 5315–5318. 10.1016/S0040-4039(01)81592-6

[B20] Strutt N. L. Zhang H. Schneebeli S. T. Stoddart J. F. (2014). Functionalizing pillar[n]arenes. Acc. Chem. Res. 47, 2631–2642. 10.1021/ar500177d 24999824

[B21] Szejtli J. (1998). Introduction and general overview of cyclodextrin chemistry. Chem. Rev. 98, 1743–1754. 10.1021/cr970022c 11848947

[B22] Wang Y. Wang C. Long R. Cao Y. Fan D. Cen M.. (2019). Synthesis and controllable self-assembly of 3D amphiphilic organoplatinum(ii) metallacages in water. Chem. Commun. 55, 5167–5170. 10.1039/c9cc02173f 30984940

[B23] Xiao T. Qi L. Zhong W. Lin C. Wang R. Wang L. (2019c). Stimuli-responsive nanocarriers constructed from pillar[n]arene-based supra-amphiphiles. Mater. Chem. Front. 3, 1973–1993. 10.1039/c9qm00428a

[B24] Xiao T. Wang J. Shen Y. Bao C. Li Z.-Y. Sun X.-Q. (2021). Preparation of a fixed-tetraphenylethylene motif bridged ditopic benzo-21-crown-7 and its application for constructing AIE supramolecular polymers. Chin. Chem. Lett. 10.1016/j.cclet.2020.10.037. [Epub ahead of print].

[B25] Xiao T. Xu L. Zhong W. Zhou L. Sun X.-Q. Hu X.-Y. (2018). Advanced functional materials constructed from pillar[n]arenes. Isr. J. Chem. 58, 1183–1193. 10.1002/ijch.201800026

[B26] Xiao T. Xu L. Zhou L. Sun X.-Q. Lin C. Wang L. (2019d). Dynamic hydrogels mediated by macrocyclic host–guest interactions. J. Mater. Chem. B 7, 1526–1540. 10.1039/C8TB02339E 32254900

[B27] Xiao T. Zhong W. Xu L. Sun X.-Q. Hu X.-Y. Wang L. (2019b). Supramolecular vesicles based on pillar[n]arenes: design, construction, and applications. Org. Biomol. Chem. 17, 1336–1350. 10.1039/C8OB03095B 30638249

[B28] Xiao T. Zhong W. Zhou L. Xu L. Sun X.-Q. Elmes R. B. P. (2019e). Artificial light-harvesting systems fabricated by supramolecular host–guest interactions. Chin. Chem. Lett. 30, 31–36. 10.1016/j.cclet.2018.05.034

[B29] Xiao T. Zhou L. Sun X.-Q. Huang F. Lin C. Wang L. (2020b). Supramolecular polymers fabricated by orthogonal self-assembly based on multiple hydrogen bonding and macrocyclic host–guest interactions. Chin. Chem. Lett. 31, 1–9. 10.1016/j.cclet.2019.05.011

[B30] Xiao T. Zhou L. Wei X. Li Z. Sun X. (2020a). Supramolecular copolymers driven by quadruple hydrogen bonding and host-guest interactions. Chin. J. Org. Chem. 40, 944–949. 10.6023/cjoc201911014

[B31] Xiao T. Zhou L. Xu L. Zhong W. Zhao W. Sun X.-Q. (2019a). Dynamic materials fabricated from water soluble pillar[n]arenes bearing triethylene oxide groups. Chin. Chem. Lett. 30, 271–276. 10.1016/j.cclet.2018.05.039

[B32] Xue M. Yang Y. Chi X. Zhang Z. Huang F. (2012). Pillararenes, a new class of macrocycles for supramolecular chemistry. Acc. Chem. Res. 45, 1294–1308. 10.1021/ar2003418 22551015

[B33] Zhang C. Li S. Zhang J. Zhu K. Li N. Huang F. (2007). Benzo-21-crown-7/secondary dialkylammonium salt [2]pseudorotaxane- and [2]rotaxane-type threaded structures. Org. Lett. 9, 5553–5556. 10.1021/ol702510c 18047364

